# Supplementary material for: Complexome profiling of the Chlamydomonas psb28 mutant reveals TEF5 as an early PSII assembly factor
Source: Plant Cell. 2025 Mar 17;37(6):koaf055. doi: 10.1093/plcell/koaf055 (PMC12164590; doi:10.1093/plcell/koaf055)
Supplement: koaf055_Supplementary_Data [file koaf055_supplementary_data.zip › psb28_interactive.zip!/plots/intensity/qualityPlots/peptides/PYR4_Cre03.g199423.t1.1.html]

### Cre03.g199423.t1.1

Trivial name: PYR4  
SeqCoverage [median]: 20.70  
Mass: 43.73 kDa  
  
 

| Sequence | MissedCleavage | Mass [Da] | wtIntensity | muIntensity | Score |
| --- | --- | --- | --- | --- | --- |
| DGFTSVADAVGADHPDIR | 0 | 1841.85 | 0.00 | 1462.94 | 49.22 |
| ELEALVNQVK | 0 | 1141.63 | 0.00 | 1336.82 | 78.33 |
| GPLGVNLGK | 0 | 853.50 | 473.36 | 1656.58 | 86.87 |
| LATHADYLVVNVSSPNTPGLR | 0 | 2223.16 | 509.88 | 2115.21 | 89.56 |
| MLDPELSHIIGIQAAAR | 0 | 1833.98 | 0.00 | 2663.11 | 89.54 |
| VAPDLNELDKR | 1 | 1268.67 | 2508.68 | 2770.24 | 48.44 |
|  |  |  | 3491.9 | 12004.9 |  |
